# Polyplexes of retinoic acid: an in vitro study of complex nanostructures against colorectal cancer cell line (HCT-15)

**DOI:** 10.1007/s10856-021-06571-1

**Published:** 2021-09-14

**Authors:** Narayan Ture, Drashti Desai, Pravin Shende

**Affiliations:** Shobhaben Pratapbhai Patel School of Pharmacy and Technology Management, SVKM’S NMIMS, V. L. Mehta Road, Vile Parle (W), Mumbai, India

## Abstract

Despite recent advances in the treatment of human colon cancer, the chemotherapeutic efficacy against colon cancer is still unsatisfactory. The complexity in colorectal cancer treatment leads to new research in combination therapy to overcome multidrug resistance in cancer and increase apoptosis. The objective of the present research work was to develop polyplexes for co-delivery of plasmid DNA with retinoic acid against colorectal cancer cell line (HCT-15). Plain polyplexes were prepared using chitosan and hyaluronic acid solution (0.1% w/v), whereas retinoic acid polyplexes were prepared using ethanol: water (1:9 v/v) system. The particle size was observed in the order of chitosan solution > blank polyplex > retinoic acid-loaded polyplex. Encapsulation efficiency of retinoic acid was found to be 81.51 ± 4.33% for retinoic acid-loaded polyplex formulation. The drug release was observed to be in a controlled pattern with 72.23 ± 1.32% release of retenoic acid from polyplex formulation. Cell line studies of the formulation displayed better cell inhibition and low cytotoxicity for the retinoic acid-loaded polyplexes in comparison to pure retinoic acid, thus demonstrating better potential action against colorectal cancer cell line HCT-15. Retinoic acid-loaded polyplexes indicated higher potential for the delivery of the active whereas the cell line studies displayed the efficacy of the formulation against colorectal cancer cell line HCT-15.

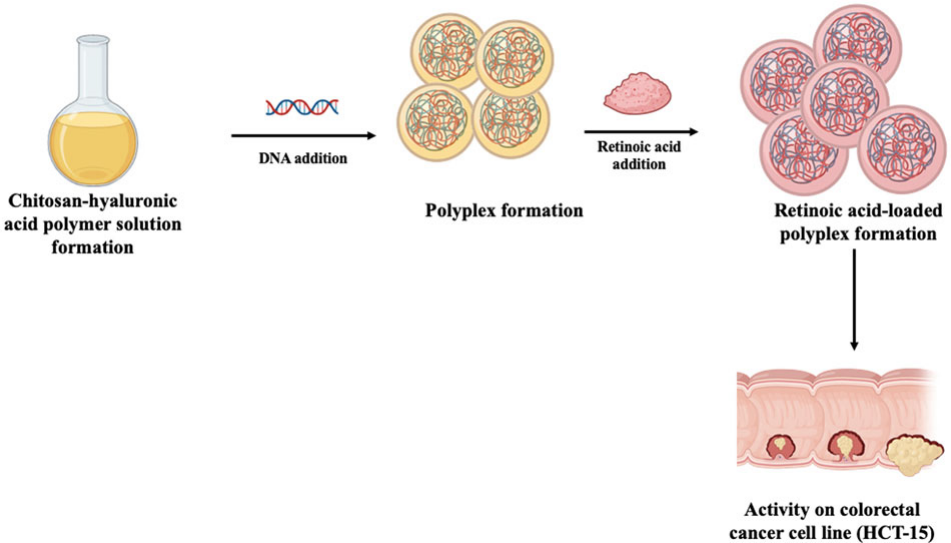

## Introduction

According to the latest statistical studies, colorectal cancer (CRC) stands third most common cancer worldwide after blood and lung cancers [1]. Numerous initiatives like theranostics and chemotherapy are mainly adopted for the treatment, but still the mortality rate of CRC is more. Chemotherapy is one of the commonly used therapies to treat cancer, however, the risk of adverse effects like nausea, vomiting, headache, alopecia, etc. is accompanied with the treatment. Recent advances in nanotechnology target cancer cells using cytotoxic drugs without affecting normal cells [2]. Over the last few years, several types of drug-loaded nanoparticulate systems like liposomes, dendrimers, polyplexes and micelles in the size range of 20–400 nm showed a strong impact for delivery of drug in chemotherapy [3]. Nevertheless, despite of high morbidity and mortality associated with CRC, the clinical development of nanoparticles for treatment remains limited. Diverse shapes, sizes and chemical natures of nanoparticles demonstrate high efficacy for encapsulating different types of anticancer therapeutics including siRNA, antibiotics, chemotherapeutics, proteins and peptides. Traditional cytotoxic agents for CRC cause several side effects like renal failure because of current therapeutic protocols based on a series of repeated administration. Recent trends in nanomedicine include the use of combined therapy of cytotoxic drugs loaded with nanocarriers to offer prolonged release, site-specific delivery and reduction or elimination of first-pass metabolism. Polymer-based nanoparticulate systems like polymer nanocapsules, polymeric mesoporous nanoparticles, nanogels, polyplexes, polymer-lipid hybrid nanosystems, polymer-drug/protein complex, polymeric dendrimers, etc. are considered for improvement of bioavailability of antineoplastic agents []. The combined therapy also includes DNA or RNA to assemble the nanoparticles for the expression or knockdown of genes to enhance the effectiveness of cytotoxic drugs like cisplatin, doxorubicin, etc. Cationic polymers such as chitosan (CS), poly(allylamine hydrochloride), polyethylenimine, etc. are commonly considered for the linkage of DNA with the polymeric matrix for enhancing the activity and stability [4]. Due to the presence of surface charges (SCs), polyplexes offer effective complexation with genetic materials viz. siRNA for gene therapy, DNA for cancer therapy, etc. Polyplexes are polymeric complex of active to increase the solubility, delay the degradation step and offer site-specific action [5, 6]. The adaptable structure enables the active to form complex linkage with polymers like polyethyleneimine and poly-l-lysine for controlled release pattern [7]. Due to proper polymeric composition and SC, polyplexes are considered as an ideal nanocarrier for the delivery of nucleic acids. Retinoic acid is a derivative of vitamin A which is present in the body that aids to inhibit CRC in mice and humans by acting on different signalling pathways for cell proliferation and growth inhibition [8]. Retinoic acid emerges as a good chemotherapeutic and chemopreventive agent because of its differentiation, antiproliferative, pro-apoptotic and antioxidant effects [9]. CRC demonstrated the presence of high levels of protein in the intestinal tissues and expressed poor degradation of retinoic acid in comparison to healthy individual. Using externally supplemented retinoic acid in the intestine or blocking the enzyme degradation significantly reduce the burden of cancer growth in the patients [10]. The objective of the present research work was to develop polyplexes for co-delivery of plasmid DNA with retinoic acid against CRC cell line (HCT-15).

## Materials and methods

### Materials

Retinoic acid was procured from Tokyo chemical industry, Japan. Plasmid DNA and hyaluronic acid were obtained from RL Chem Industries, India. HCT-15 human cell line was purchased from National Centre for Cell Sciences, Pune, India. All other reagents were of analytical grade and used without further purification.

### Methods

#### Formulation of chitosan (CS)-hyaluronic acid (HA) polyplexes

0.02% W/v CS solution was prepared with 0.1 M acetic acid by maintaining the pH 3.5 and sodium sulphate solution (5.0 mM) was individually pre-heated at 60 °C. CS solution of equal volume was added to sodium sulphate solution; the solution was vortexed for 30 s and submerged in an ice bath to maintain the temperature at 5 °C. 0.1% w/v HA solution was added to 5 mM sodium sulphate and CS mixture. To formulate CS and CSHA polyplexes with pDNA, ratio of PO_4_: NH_3_ group as 1:5 of CS and pDNA was used (Table 1). pDNA was added to sodium sulphate and CS solutions for the formation of polyplexes [11]. The blank CS polyplexes were prepared by the same procedure described above without addition of retinoic acid.

**Table 1:** Compositions of different formulations of polyplexes

	Formulation			
Ingredient	P1	P2	P3	P4
Hyaluronic acid	1 mg	1 mg	1 mg	1 mg
Chitosan	2 mg	2 mg	2 mg	2 mg
Sodium sulphate	0.7 g	0.7 g	0.7 g	0.7 g
DNA	–	–	50 µg	50 µg
Retinoic acid	–	1 mg	–	1 mg

#### Preparation of retinoic acid polyplexes

The retinoic acid was added in ethanol: water system (1:9 v/v) and subsequently mixed with sodium sulphate solution. Finally, CS and retinoic acid solutions were mixed and vortexed for 50 s for the formation of retinoic acid-loaded polyplexes [12] and abbreviated as P1, P2, P3 and P4 (Table 1).

### Characterisation of polyplexes

#### Particle size and SC

The measurements of particle size and SC of polyplex (formulations P1–P4) were obtained by dispersing the samples in distilled water at 25 °C using Malvern Zetasizer (Nano ZS90, UK) [13].

#### % Encapsulation efficiency (EE)

Retinoic acid-loaded polyplexes formulations (P2 and P4) were accurately weighed and centrifuged in 10 mL measuring tubes for 20 min at 5000 rpm. The supernatant was collected and estimated for absorbance at 349 nm using UV–Visible spectrophotometer (Shimadzu, Japan).

The % EE was calculated using following formula:

$$\begin{array}{l}\% \,{{{{{\mathrm{EE}}}}}} \,=\, \left( {{{{{{\mathrm{Total}}}}}}\,{{{{{\mathrm{amount}}}}}}\,{{{{{\mathrm{of}}}}}}\,{{{{{\mathrm{retinoic}}}}}}\,{{{{{\mathrm{acid}}}}}}\,{{{{{\mathrm{in}}}}}}\,{{{{{\mathrm{polyplexes}}}}}}} \,-\, \right.\\ {{{{{\mathrm{Amountofretinoic}}}}}}\,{{{{{\mathrm{acid}}}}}}\,{{{{{\mathrm{in}}}}}}\,{{{{{\mathrm{the}}}}}}\,{{{{{\mathrm{supernatant}}}}}}/\\ \left. {{{{{{\mathrm{Total}}}}}}\,{{{{{\mathrm{amount}}}}}}\,{{{{{\mathrm{of}}}}}}\,{{{{{\mathrm{retinoic}}}}}}\,{{{{{\mathrm{acid}}}}}}\,{{{{{\mathrm{in}}}}}}\,{{{{{\mathrm{polyplexes}}}}}}} \right) \,\times\, 100.\end{array}$$

#### In vitro release study

The polyplex formulations P2 and P4 were placed individually in a previously soaked dialysis membrane (Himedia, India). After adding formulation to dialysis bag, it was tied from both the ends and placed in 30 mL of phosphate buffer saline pH 7.4 on a magnetic stirrer with a speed of 150 rpm at 37 °C. Aliquots were withdrawn at pre-determined time intervals of 1, 2, 4, 8 and 24 h and sink condition was maintained with fresh phosphate buffer saline solution of pH 7.4. Aliquots were analysed using UV–visible spectrophotometer at the wavelength 349 nm [14].

*Based on the particle size, SC, % EE and in vitro release studies, formulations P3 and P4 were considered for further characterisation.*

#### Transmission electron microscopy (TEM)

The morphological examinations of polymeric solution and polyplex formulations P3 and P4 were performed by TEM (JEOL JEM-1011 electron microscope). The freshly prepared formulations were placed on copper grids for few minutes, then air-dried and stained with phosphotungstic acid (2% w/v) before examination.

#### FTIR spectroscopy

FTIR spectra of HA, retinoic acid and formulations P3 and P4 were analysed using potassium bromide (KBr) pellet method. The samples were analysed from 400 to 4000 cm^−1^ at 25 °C with 32 scans using FTIR spectroscopy (Perkin Elmer, USA).

#### Gel electrophoresis

HA/CS-plasmid DNA polyplex formulations P3 and P4 were analysed using agarose gel electrophoresis. The polyplex formulations and naked plasmid DNA (average size 2 kb) were loaded onto 1% agarose gel with ethidium bromide in Tris-borate EDTA buffer at pH 8. The formulations P3 and P4 were placed on the gel at 120 V for 30 min and photographed using Alpha Innotech, USA [15].

#### In vitro cell cytotoxicity study

In vitro studies for drug and formulations P1 to P4 on CRC cell lines HCT-15 were carried out by inoculating in 96-well microtiter plates with a capacity of 100 µL using MTT assay. The plate was supplemented with DMEM and 10 % FBS along with samples dissolved in DMSO (100 mg/mL), diluted further to achieve 1 mg/mL using distilled water to obtain a serial concentrations of 100 μg/mL, 200 μg/mL, 400 μg/mL, 800 μg/mL and aliquots of 10 µL and added later to the microtiter wells of 90 µL of medium and the final active concentration was of 10 μg/mL, 20 μg/mL, 40 μg/mL and 80 μg/mL. The plate was incubated at 37 °C with 5% CO_2_, 95% air and 100% RH for 24 h. The plate was read at 570 nm to obtain the absorbance by conversion of MTT to formazan used to calculate the cell growth concentrations using given formula [16–19].

$$\begin{array}{l}\% {{{{{\mathrm{Growth}}}}}}\,{{{{{\mathrm{inhibition}}}}}} \,=\, \left( {{{{{{\mathrm{Different}}}}}}\,{{{{{\mathrm{concentrations}}}}}}\,{{{{{\mathrm{of}}}}}}\,} \right.\\ {{{{{\mathrm{test}}}}}}\,{{{{{\mathrm{growth}}}}}}\,{{{{{\mathrm{in}}}}}}\,{{{{{\mathrm{the}}}}}}\,{{{{{\mathrm{presence}}}}}}\,{{{{{\mathrm{of}}}}}}\,{{{{{\mathrm{different}}}}}}\,{{{{{\mathrm{drug/Concentration}}}}}}\\ \left. {{{{{{\mathrm{of}}}}}}\,{{{{{\mathrm{control}}}}}}\,{{{{{\mathrm{growth}}}}}}} \right) \,\times\, 100\% \end{array}$$

## Results

### Particle size and SC

The particle size and SC of formulations P1 to P4 are shown in Table 2 where the polyplexes (formulations P1–P4) were found to be in the range between 284.9 ± 31.23 nm to 526.4 ± 94.25 nm. Polyplex formulations P1 and P2 without plasmid DNA showed the particle size 284.9 ± 31.23 nm and 427.35 ± 40.57 nm, respectively, while those preparations formulated with plasmid DNA in the form of formulations P3 and P4 revealed the size of 341.5 ± 70.45 nm and 526.4 ± 94.25 nm, respectively. The particle size of the polyplexes depends on the molecular weight of the anionic polymer and it also influences the morphology of polyplexes structures. The zeta potential of polyplexes showed cationic charges in the range of 15.23 ± 5.47 mV to 24.37 ± 4.32 mV and indicated better colloidal stability [20]. The particle size showed strong interaction of CS with negatively charged plasmid DNA to increase the large chain entanglement and produce nano-dimensional polyplexes. An average PDI value below 0.3 represented homogeneous distribution of all the formulations of polyplex. Slight increase in particle size and decrease in SC were observed due to the presence of HA.

**Table 2:** Particle size and SC of formulations P1 to P4

Formulation	Particle size (nm ± S.D.)	SC (mV ± S.D.)	PDI
P1	284.9 ± 31.23	15.8 ± 0.14	0.273
P2	427.35 ± 40.57	21.7 ± 0.23	0.266
P3	341.5 ± 70.45	19.35 ± 0.58	0.245
P4	526.4 ± 94.25	18.27 ± 0.47	0.160

### % EE

**Fig. 1:**
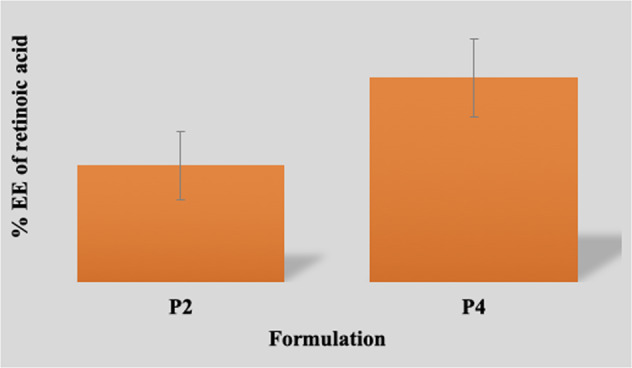
% EE of retinoic acid-loaded polyplex formulations. Data were analysed using Student’s *t* test (*p < 0.05) when compared with polyplex formulation without pDNA

Formulations P2 displayed % EE of retinoic acid of 72.25 ± 3.42%, whereas formulation P4 showed 81.51 ± 4.33%, and statistically analysed using Student’s *t* test with significant values on addition of pDNA (Fig. 1). Thus, loading of retinoic acid depends on the ratio of drug and polymer possessing positive effect. The presence of CS in polyplexes provided high encapsulation efficiency of DNA whereas the encapsulation of drug into polyplex structure showed an association of cations of chitosan and anions of drug molecules. Moreover, hydrophobic interactions or hydrogen bonds between the organic bases of the nucleotide and the sugar structure of polymer played important role in the stability. These results supported the hypothesis of multiple interactions between CS and pDNA.

### In vitro release study

**Fig. 2:**
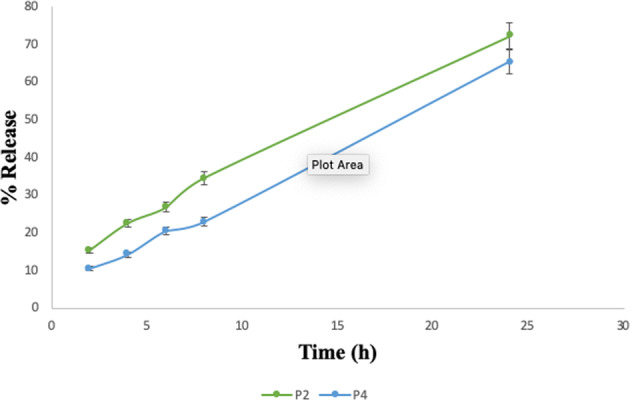
% Drug release profiles of retinoic acid from polyplex formulations P2 and P4

The formulation P2 displayed highest % retinoic acid release of 72.23 ± 1.32% followed by P4 with the release of 65.43 ± 3.14% for 24 h (Fig. 2). The in vitro study displayed maximum % release of retinoic acid within 2 h, even though formulation P2 showed prolonged release for 24 h of retinoic acid due to its complexation with polymer. The difference in efficiency of percentage drug release by formulations P2 and P4 was not statistically significant as per Student’s *t* test (*p* value < 0.05) [21]. This might be due to the strong interaction of CS with negatively charged pDNA in formulation P4 whereas in formulation P2, pDNA was not present.

### TEM

**Fig. 3:**
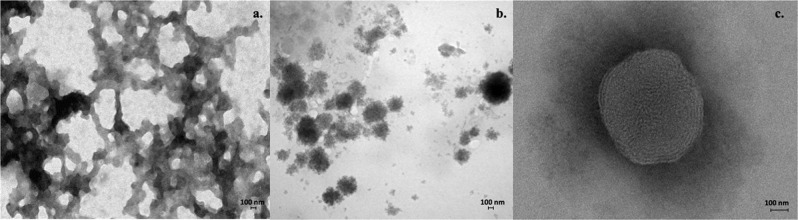
TEM of (**a**) polymer solution, (**b**) formulation P3 and (**c**) formulation P4

TEM showed that formulations P3 and P4 formed heterogeneous populations of spherical, toroidal and oblong polyplexes. The TEM studies displayed that the formulation P3 showed aggregation of particles due to the incorporation of the HA into CS polyplex structure (Fig. 3).

### FTIR spectroscopy

**Fig. 4:**
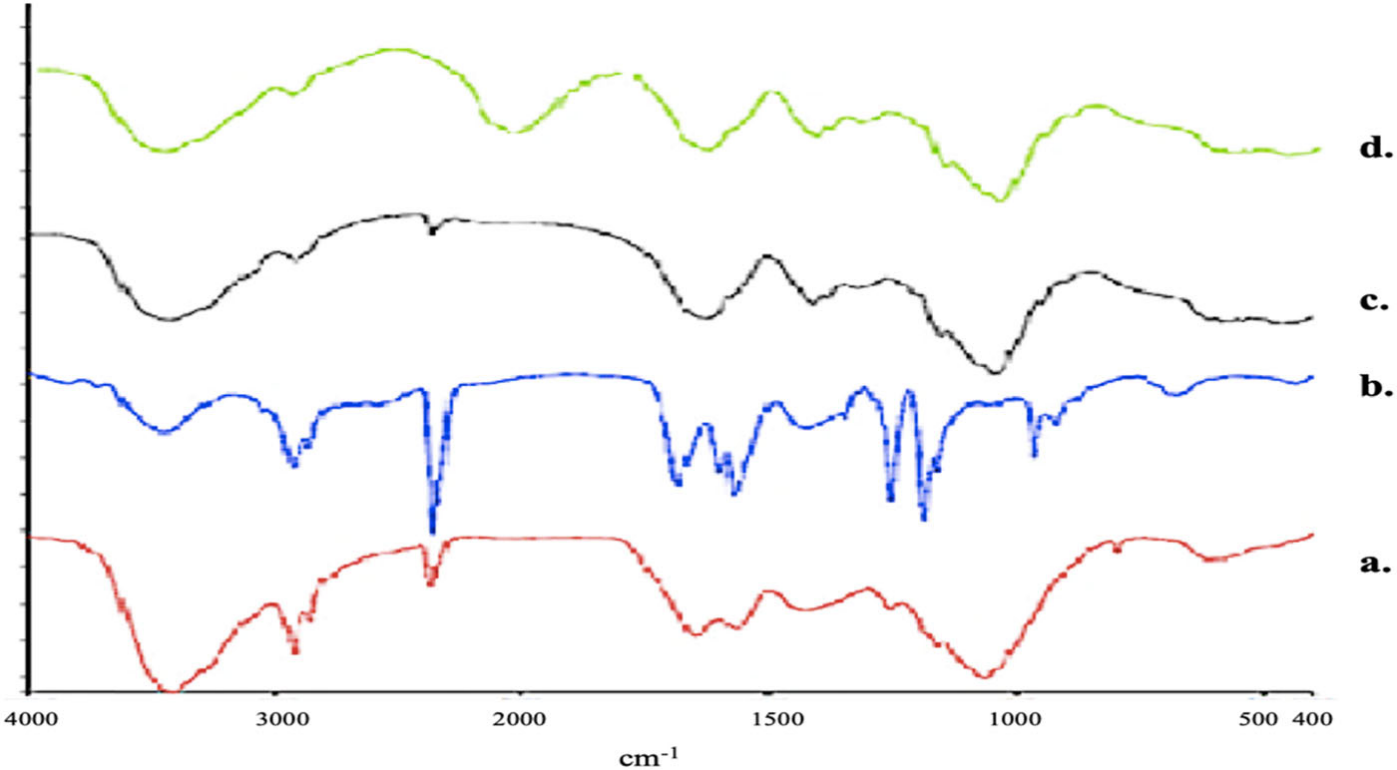
FTIR spectra of (**a**) hyaluronic acid, (**b**) chitosan solution and formulations, (**c**) P3 and (**d**) P4

FTIR spectra confirmed the formation of polymer-DNA complex and drug-loaded polyplexes. FTIR spectrum of formulation P4 was compared with the retinoic acid as shown in Fig. 4. The spectrum of blank polyplex formulation (P3) exhibited the presence of CS and HA in the region of 3291–3161 cm^−1^ corresponds to the N-H and O-H stretching. The peaks at 1720 and 1648 cm^−1^ corresponded to carbonyl stretching of carboxylic acid and amide, respectively and indicated the presence of HA in the formulation. In FTIR studies, the major peaks at 1800–1500, 1500–1250 and 1250–800 cm^−1^ in three regions confirmed the presence of DNA in the formulation P4.

### Gel electrophoresis

**Fig. 5:**
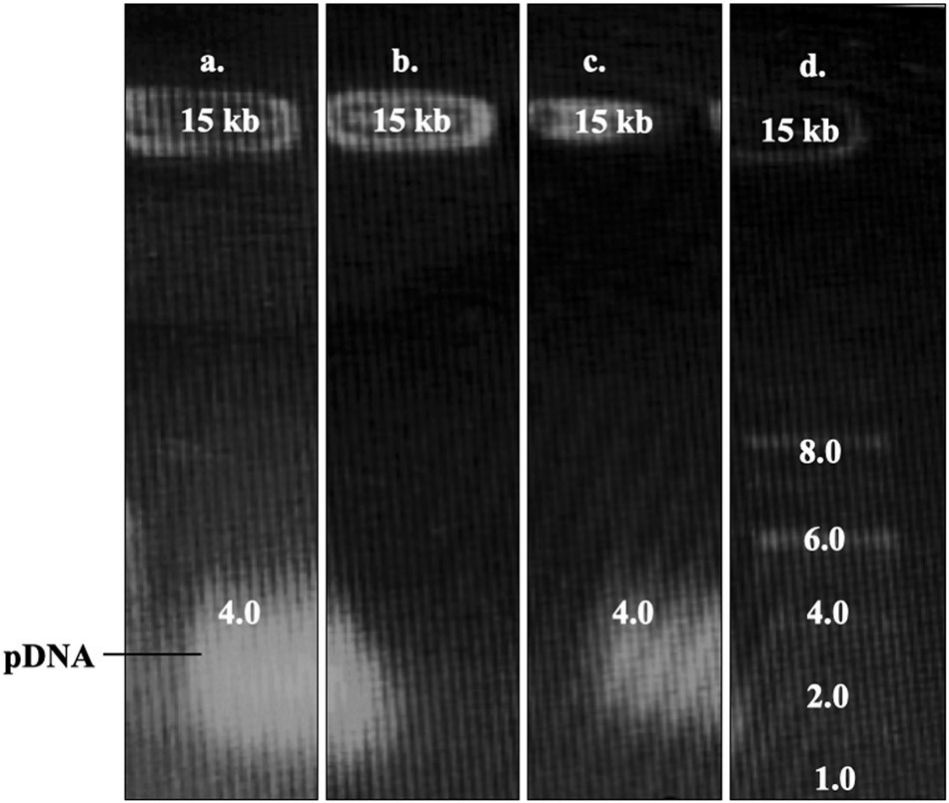
Gel electrophoresis of (**a**) plasmid DNA, (**b**) formulation P3, (**c**) formulation P4 and (**d**) standard marker

The complexation and retention of plasmid DNA by polyplex formulations were evaluated using gel electrophoresis. In agarose gel, formulations P3 and P4 at all concentrations showed no migration of pDNA signal at 2 kb, indicating the effective and complete complexation with the polymer. All the formulations showed complexation with pDNA whereas absence of pDNA migration was observed in Fig. 5 [22, 23]. There was no other free DNA band displayed, indicating a steric interaction between polymer and plasmid DNA [24, 25].

### In vitro cell cytotoxicity study

For the assessment of cytotoxic action, formulations P1, P2, P3 and P4 were treated on HCT-15 cell lines using pDNA at collective N/P ratios present in polyplex formulations (2.5, 5, 7.5, 10 and 12.5) where retinoic acid showed IC_50_ value of 5 and formulations P1, P2, P3 and P4 depicted IC_50_ values of 2, 6, 3.5 and 3, respectively at the concentration of 10 μg/mL [26]. As the formulation P1 contained no DNA or drug, it’s IC_50_ value was found to be the lowest whereas formulation P3 contained pDNA which showed a minimal cytotoxic effect. On the other hand, the content of drug (retinoic acid) was more in formulation P2 compared to the mixture of DNA and retinoic acid in formulation P4, hence it showed reduction in the IC_50_ value [27] .The percentage inhibition of HCT-15 cells was conducted on plain retinoic acid and formulations P1 to P4. The % inhibition was highest for P4 i.e. 99.02% ± 0.024% and for formulation P2, it was found to be 92.43% ± 2.52% at 12.5 N/P ratio (Table 3). The reported dose of retinoic acid is 60–90 mg per day and more doses lead to adverse side effects like nausea, flaking skin, dry mouth, etc. but the use of polyplexes formulation displayed dose reduction and site-specific action to the cancerous cells resulting in lower toxic effects as reported by Estevis and Varshosaz [28, 29]. Formulation P4 showed better cell inhibition at lesser dose (10 μg/mL) in comparison to retinoic acid for the treatment against HCT-15 cell line. Moreover, the formulation P2 contained 15 µg/mL of the drug which was higher than formulation P4 (10 µg/mL) consisting of drug and excipients with pDNA for providing the synergistic effect, leading to better cell inhibition.

**Table 3:** ll inhibition by drug and different formulations P1, P2, P3 and P4 at different concentrations

Sample	N/P ratio	% Cell inhibition			
		1	2	3	Average (%±S.D.)
Retinoic acid	15 µg/mL	54.2	51.01	52.1	52.44 ± 1.32
Formulation P1	2.5	3.76	4.51	2.92	3.73 ± 0.65
	5	7.43	9.11	6.34	7.63 ± 1.14
	7.5	10.36	8.05	13.44	10.62 ± 2.21
	10	13.75	10.97	12.51	12.41 ± 1.14
	12.5	16.03	14.08	17.16	15.76 ± 1.23
Formulation P2	2.5	72.13	68.41	74.62	71.72 ± 2.55
	5	76.73	78.63	75.15	76.84 ± 1.42
	7.5	81.48	84.39	79.03	81.63 ± 2.19
	10	89.23	86.25	91.06	88.85 ± 1.98
	12.5	95.11	89.05	93.13	92.43 ± 2.52
Formulation P3	2.5	10.16	12.67	9.04	10.62 ± 1.51
	5	15.78	18.02	17.93	17.24 ± 1.03
	7.5	21.44	27.61	25.27	24.77 ± 2.54
	10	39.07	37.13	32.36	36.19 ± 2.81
	12.5	37.43	51.47	42.24	43.71 ± 5.82
Formulation P4	2.5	82.33	78.16	81.42	80.64 ± 1.79
	5	86.07	88.14	84.52	86.24 ± 1.48
	7.5	91.54	94.06	93.27	92.96 ± 1.05
	10	95.24	92.35	97.06	94.88 ± 1.94
	12.5	98.99	99.01	99.05	99.02 ± 0.024

## Discussion

On the basis of evaluations tests like particle size, % EE and in vitro drug release, formulation P3 without retinoic acid and formulation P4 containing retinoic acid, CS, HA, sodium sulphate and DNA were found to be suitable against CRC cell line HCT-15. Cross-linking reaction between HA and CS helped to slow the release of retinoic acid for 6 h. Gel electrophoresis confirmed the complexation of pDNA in the form of polyplexes by demonstrating no migration in the band. The in vitro cell cytotoxicity study indicated that polyplexes displayed significant dose reduction in comparison to the recommended dose of retinoic acid for therapeutic application with significant cell inhibition. Hence, it is resulted that retinoic acid possesses the activity against HCT-15 cell growth at higher concentration (15 μg/mL) but on incorporation within polyplexes it showed similar inhibition activity on the HCT-15 cell line. This indicated better anticancer action at reduced concentration of retinoic acid by charge neutralisation of pDNA as well as polymer structure aiding the management of CRC by significant penetration of the polyplexes into the cell. Thus, the polyplex formulation inhibited the cancer growth and proposed for potential application in CRC.

## Conclusions

The retinoic acid-loaded polyplexes were successfully formulated against CRC cell line HCT-15. The polyplex formulations were characterised by several parameters like particle size, SC, drug encapsulation, in vitro, gel electrophoresis and in vitro cell cytotoxicity studies. The network structure of the polymer solution and drug-loaded polyplex in the TEM analysis verified the formation of polyplex structure. Thus, the polyplexes act as a promising delivery against cancer cell line HCT-15 and also suggest as a new carrier in cancer therapy.
